# Smoking and the occurrence of larynx cancer in Sweden – a population analysis

**DOI:** 10.1177/14034948251327872

**Published:** 2025-03-21

**Authors:** Bengt Järvholm, Per Liv, Linnea Hedman, Maréne Landström, Kjell Torén, Alex Burdorf

**Affiliations:** 1 Department of Public Health and Clinical Medicine, Umeå University, Umeå, Sweden; 2 Pathology Section, Department of Medical Biosciences, Umeå University, Umeå, Sweden; 3 Department of Public Health and Community Medicine, Sahlgrenska Academy, University of Gothenburg, Gothenburg, Sweden; 4 Department of Public Health, Erasmus MC, Rotterdam, the Netherlands

**Keywords:** Asbestos, epidemiology, tobacco, smoking

## Abstract

**Aims:**

To study the importance of decreasing tobacco smoking on the occurrence of larynx cancer in men and women.

**Methods:**

The incidence rates of larynx cancer in the Swedish population between 1970 and 2021 were retrieved from the Swedish Cancer Register for ages 50–84 years, stratified for sex, age and calendar year. Data on the population’s smoking habits was retrieved from surveys and from taxation on the sale of cigarettes. The occurrence of larynx cancer was compared to smoking habits, sex and age. The time trends were compared between larynx and lung cancer.

**Results:**

Over the years, Swedish men and women have had different smoking habits, especially older persons during the 1970s. In 1963, the prevalence of current smokers in women 50–69 years was 11%, while it was 46% in men. Around 2020, less than 10% of men and women in all age groups were current smokers. However, men had higher incidence rates of larynx cancer than women, even when their smoking habits were similar. For example, men and women 60–64 years of age in 2017–2021 had similar smoking habits during their life but the relative risk of larynx cancer in men compared to women was 3.3 (95% CI 1.7–4.8). However, pipe smoking was much more common in men.

**Conclusions:**

**The study indicates that other causes than cigarette smoking have an impact on the occurrence of larynx cancer in Sweden. Pipe smoking and occupational exposure to carcinogenic materials such as asbestos may be underlying causes of the difference in cancer risk between Swedish men and women.**

## Background

Larynx cancer is a relatively rare type of cancer, typically classified as a squamous cell carcinoma. For instance, there were a total of 199 registered cases of larynx cancer in the Swedish Cancer Register in 2021, which constituted around 0.3% of all registered cases of cancer. It is more common in men than in women: 158 cases occurred in men and 41 in women in 2021. Tobacco smoking is a significant cause of larynx cancer [1]. The International Agency for Research on Cancer (IARC) concluded in a review that it is one of the cancers most strongly associated with smoking and that the risk decreases quite quickly after cessation of smoking. They noted that ‘No detectable higher risk compared with never-smokers was seen among subjects who had quit smoking for at least 10 years’ [2]. A study in central Europe attributed 87% of the cases to smoking: 75% to current smoking and 12% to former smoking [3]. Alcohol is also considered a risk factor but of lower relative risk than smoking [2, 4]. However, there does seem to be a positive interaction between smoking and alcohol intake [5].

In a review from 2012, IARC classified exposure to asbestos as a cause of larynx cancer [6]. Two Swedish studies found that workers occupationally exposed to asbestos had double the relative risk of developing larynx cancer [7, 8]. However, a review in 2016 based on 15 studies concluded that current evidence lacked support for an association between asbestos and larynx cancer [9]. Ten of the studies showed no correlation between asbestos exposure and the other studies had limited adjustments for confounders.

In Sweden, the proportion of smokers in the population have decreased and the prevalence in 2021 was around 3%–8% according to national surveys [10, 11]. In a previous study on the occurrence of lung cancer and smoking in Sweden we found a decreasing trend in squamous cell lung cancer but an increasing trend of adenocarcinomas, suggesting that the association between smoking and lung cancer differs across histological cell type [12]. The objective of the current research was to estimate the effect of decreasing smoking habits on the occurrence of larynx cancer. Furthermore, we wanted to compare the occurrence of lung cancer and larynx cancer, two types of cancer strongly related to tobacco smoking.

## Methods

The number of cases of larynx cancer (ICD 7 code 161) and lung cancer (ICD 7 code 161) stratified for calendar year, age (5-year age groups), cell type and sex were collected from the Swedish Cancer Register from 1970 to 2021. Data about the corresponding size of the population for the strata were obtained from Statistics Sweden.

More than 95% of the cases were classified as squamous cell cancers and we restricted the analysis to this histological cell type. Larynx cancer typically appeared between the age of 50 and 84. Few cases were younger than 50 years of age, 9% in 1970 and 3% in 2021; persons aged 85 years and older constituted 1% in 1970 and 8% in 2021. Very few elderly persons (85+) have been included in the smoking surveys and their age distribution varies by calendar year. Therefore, the analysis was restricted to ages 50–84 years.

We also compared the occurrence of lung cancer in the population with the occurrence of larynx cancer using data from the Swedish Cancer Register using the same time period, age groups and cell types.

Smoking habits were estimated through the sale of cigarettes and from national surveys. Surveys became more frequent in the mid-1970s, therefore, we restricted the analysis to 1970 and later. At the time of analysis, the last year of data from the Cancer Register was 2021.

Sex- and age-stratified time trends of cancer incidence were estimated by Poisson regression models using the GENMOD procedure in SAS^©^. The time trend for the period was estimated as ln(rate)=A*year + B, where A (the slope) and B are constants (see Table S1). Wald estimates were used to calculate the confidence intervals. The incidence rate ratios between men and women were estimated by Poisson regression with sex as dependent variable, stratified by age group and across 5-year calendar intervals (see Tables I and S2).

**Table I.** Comparison of incidence rates of larynx cancer between men and women.

Age group	Year		
	2000 (1998–2002)^a^	2010 (2008–2012)	2019 (2017–2021)
	RR (95% CI)^b^	RR (95% CI)	RR (95% CI)
50–54	4.0 (2.3–7.0)	2.7 (1.3–5.6)	3.3 (1.5–7.2)
55–59	4.2 (2.6–6.9)	5.0 (2.8–8.8)	2.8 (1.7–4.8)
60–64	5.4 (3.4–8.6)	5.1 (3.3–7.7)	3.3 (2.1–5.3)
65–69	6.0 (3.7–9.7)	5.8 (3.9–8.6)	3.2 (2.2–4.7)
70–74	8.0 (5.0–12.8)	4.2 (2.8–6.4)	3.6 (2.5–5.1)
75–79	5.7 (3.7–8.8)	5.1 (3.0–8.5)	7.1 (4.5–11.1)
80–84	13.7 (6.6–28.5)	8.8 (4.6–16.6)	14.1 (6.5–30.5)

a Model ln(rate) = A*sex + B .

b Relative risk (RR) with women as reference; 95% confidence intervals (CIs) in parenthesis.

## Results

**Figure 1.**
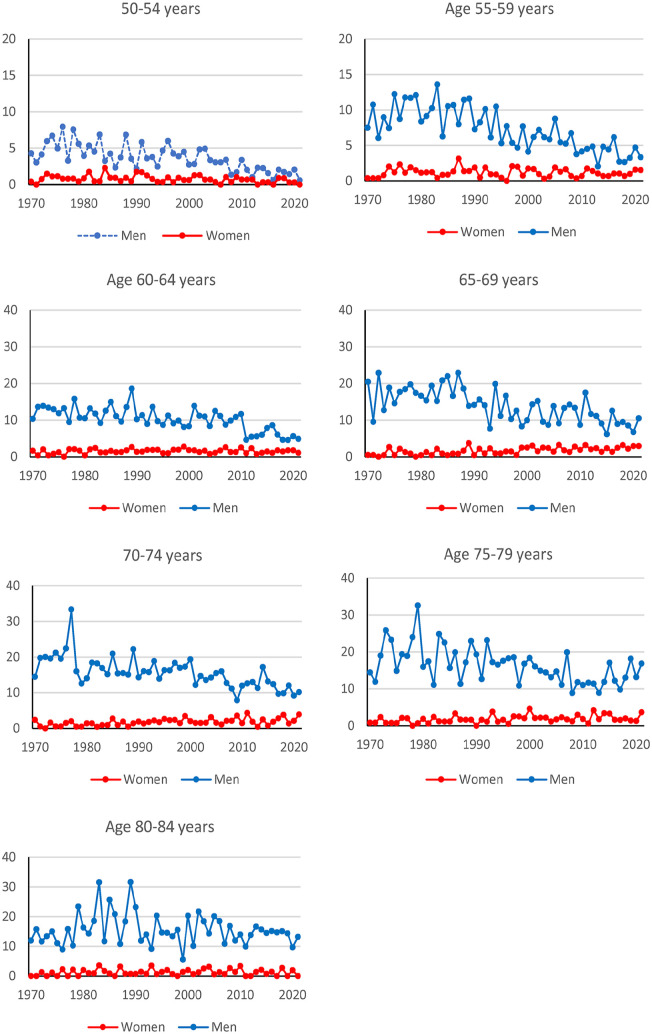
Incidence rates for squamous cell larynx cancer (cases per 100,000 person-years) according to age, sex and time. Note the differences in scales on the y-axes.

Cases from 1970 to 2021 comprised 7603 males and 1121 females. The incidence rates were higher in men than women during the whole period (Figure 1). Typically, there were fewer than five cases per year and age group among women, while corresponding numbers among men were above 10 cases and often 20–40 cases. In the youngest age groups, the rates in men approached the rates among women. The incidence rates in men decreased over time in ages below 80 years (Supplementary Table S1). The rates increased in women 65–79 years old, but the estimates have low precision due to the few cases.

A comparison of the incidence rates between the sexes in around 2000, 2010 and 2019 shows that while the rates became more similar in the younger ages, there was no such trend among those aged 75–84 (Table I). The relative risk in the earlier periods are described in the Supplemental material (Table S2).

**Figure 2.**
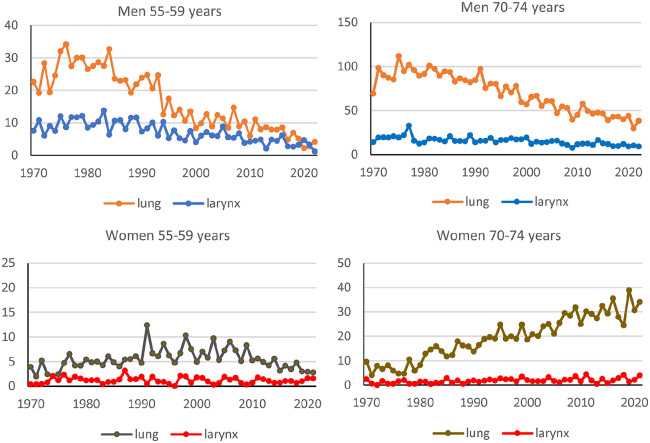
Incidence rates (cases per 100,000 person-years) for squamous cell lung cancer and squamous cell larynx cancer according to sex, age (55–59 and 70–74) and calendar year between 1970 and 2021. Figures for age groups 50–54, 60–64, 65–69, 75–79, and 80–84 years can be found in Supplemental material (SF1).

Smoking is a well-established risk factor for lung cancer, especially squamous cell lung cancer. Therefore, we compared the occurrence of cancer in the lungs and larynx by age. The differences in incidence rates were higher in the 1970s and 1980s for all age groups among men (see Figure 2, and SF1 in Supplemental material). For ages 50–59 years, there were small differences in incidence rates between squamous cell lung cancer and squamous cell larynx cancer in men and women in 2010–2021. Women aged 65–84 had a different pattern than men of the same age, with similar rates in the early period and high differences in 2010–2021. Men had a statistically significant (*p* < 0.05) decreasing trend for larynx cancer in all age groups except the oldest, 80–84 years. Women had a statistically significant increasing trend for larynx cancer in age groups 65–69 and 75–79 years (*p* < 0.05) (see Figure 2 and Table S5).

A comparison between adenocarcinoma in the lungs and squamous cell larynx cancer in men showed quite different time trends, with an increasing trend for adenocarcinoma in lung cancer (see SF2 in Supplemental material).

### Smoking

**Figure 3.**
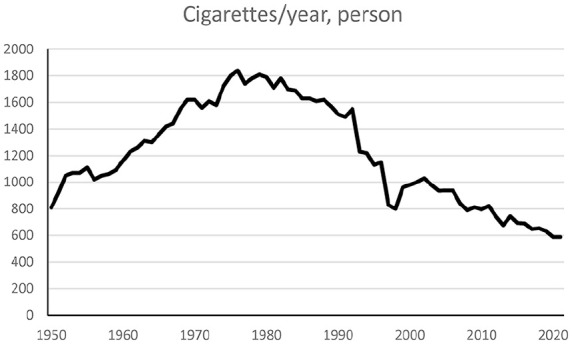
Cigarettes per year and person over 15 years of age according to taxation [13–15].

Data from taxation of tobacco showed that the sale of cigarettes per person 15 years and older increased from the 1950s to around 1980 and then decreased (Figure 3). The number of cigarettes per person and year increased from around 800 in 1950 to around 1800 in 1980 and then decreased to around 600 in 2021.

Survey data have mainly registered current smoking habits and reported findings for different age groups over time. Very few have included the data of persons of a more senior age. A large survey in 1963 of 55,000 persons found that 46% of men and 11% of women aged 50–69 were current smokers [13]. In a survey in 1980 of persons 65–74 years of age, 32% of the men and 14% of the women reported that they were current smokers, while the figures in 2000 for the same age group were 16% and 15% for men and women, respectively [15]. The prevalence of current smokers among men and women 65–84 years was 11% and 12% in 2004 and 7% and 8% in 2021 [16]. Analyses have indicated that the frequency of current smokers among men and women in cohorts born in 1940 and the 1950s had similar smoking habits in 1988/89 and 2004/5 [17]. A comparison of the prevalence of current smokers in older men and women is described in the Supplemental material (Tables S3 and S4).

Pipe smoking was previously quite common among men. In the early 1970s, 23%–26% of men reported that they smoked a pipe at least every week [13], but the prevalence had decreased to around 11% in the early 1980s. Pipe smoking has always been far less common among women: in the late 1970s and early 1980s the prevalence was around 1% [13]. Data from taxation indicated that pipe smoking has further decreased. Tobacco products for the pipe, cigars and cigarillos constituted 6% of sold tobacco products in Sweden in 2010 [18].

## Discussion

The analyses showed decreasing trends between squamous cell lung cancer and squamous cell larynx cancer in men of all ages, indicative of the importance of tobacco smoking as a cause of larynx cancer as smoking habits have decreased in men. However, the incidence rates have not decreased as much as was predicted by IARC [2]. There was a large difference in incidence rates for larynx cancer among men and women even in periods when their smoking habits were similar, which indicates differences in causes. However, similarities in female time trends for squamous cell lung cancer and squamous cell larynx cancer indicated that tobacco smoking is also a cause of female larynx cancer.

### Methodological aspects

Measuring tobacco smoking is often done by surveys. However, participation in surveys in Sweden has decreased over time. Furthermore, the questions, age groups included and areas sampled have also varied [12]. A survey by Statistics Sweden in 1963 about smoking habits with around 55,000 randomly selected participants had a participation rate of 87% from the postal survey (from three separate mailings), which increased to 93% by supplementing this with interviews [19]. Recent national surveys of around 40,000 persons have garnered participation rates of around 44% [16]. There are indications that smokers have a lower participation rate in such surveys [20]. A comparison of cigarette sales and number of cigarettes smoked also indicates that smokers are underrepresented among participants [12]. The smuggling of tobacco may cause underestimations of consumption, but estimates of smuggling indicate that the amount is modest [21]. Furthermore, smokers may stop and start the habit several times and vary in consumption over time. A comparison between cigarette sales (Figure 3) and survey data indicated that the survey data may underestimate the prevalence of smokers [12]. However, these difficulties are unlikely to explain the large differences in the occurrence of larynx cancer in men and women.

This is an ecological analysis as we lacked data on individual smoking habits, which is a weakness of the study. However, the Swedish Cancer Register and data on the sales of cigarettes include groups that can be difficult to both reach and involve in studies of random samples of the population, such as persons with addictions or mental disorders. The national incidence rates are from a dynamic population in which some are immigrants and some emigrate, however, this migration is unlikely to have any major impact on our findings, especially not on the comparison between the occurrence in lung and larynx cancer and in older ages [12].

### Smoking as a cause of larynx cancer

There is a correlation between the occurrence of larynx cancer and squamous cell lung cancer, which is a type of cancer that is strongly related to smoking. This correlation supports the significance of smoking as a cause of male larynx cancer. The weak increase in the occurrence of female larynx cancer with increasing female smoking habits indicates that the amount of smoking among Swedish women so far has had far less impact on the occurrence of larynx cancer than smoking among men has. The low number of occurrences among women makes studying exposure–response relationships from population data difficult. The difference could depend on the co-occurrence of other factors in men that promote the effect of tobacco smoke. A case referent study found a non-significant effect of alcohol alone (OR 1.21, 95% CI 0.77–1.92), a strong effect of tobacco alone (OR 6.76, 95% CI 4.58–9.96) and an even stronger effect of tobacco and alcohol together (OR 14.22, 95% CI 8.26–24.46) [5]. The joint population-attributable fraction was estimated at 88.5%. Only 1.6% of the cases with larynx cancer were reported as non-smokers and not using alcohol, while the corresponding figure was 14.9% for the referents.

The alcohol consumption among Swedish men has consistently been higher than among women. In 2004–2021, men 65–84 years of age on average consumed about twice as much as women of the same age, that is, 4.5 and 2.2 L per year, respectively [22]. The total procurement of alcohol to residents 15 years and older has been fairly stable over the period 2001–2021 and between 8.5 and 10.6 L/year [23]. We are not aware of any data on the proportion of men and women that are heavy smokers that also are heavy drinkers in Sweden. However, we think that the very large difference between men women, especially at higher ages, is hard to attribute only to a difference in smoking and drinking habits.

Pipe smoking was previously common among men but rare among women and may be a cause of the difference in occurrence of larynx cancer between men and women. The Swedish habit of smoking a pipe in the 1900s differed from smoking a waterpipe as the smoke is directly inhaled from highly heated tobacco, while the smoke in waterpipes passes through water before it is inhaled [24]. We did not find any studies of the composition of smoke from Swedish pipe smoking. ‘Swedish’ pipes contain no filters and there is often a viscous oil in the pipe that may contain chemicals with high boiling points, for example, polycyclic aromatic hydrocarbons. Sucking on the pipe might cause inhalation of an aerosol of the oil, with potentially large droplets that could accordingly be deposited in the proximal parts of the respiratory system such as the larynx.

### Other causes

A possible cause of the difference in incidence rates of larynx cancer between men and women is occupational exposure. The interaction between tobacco smoking and dust and other pollutants is well known, for example, the interaction between asbestos and tobacco smoking in the cause of lung cancer [25, 26]. A systematic review indicated increased risks of larynx cancer for occupational groups exposed to air pollutants in their jobs, while workers in professional or technical jobs had a lower risk [27]. Men and women have traditionally held different types of employment and worked in different sectors in Sweden, the same is true today. Men work more often in manufacturing and construction industries, while women work in health and social care. The proportions of women in the Swedish construction industry were 3%, 6% and 7% in 1960, 1975 and 2005, respectively [28, 29, 30]. Two previous Swedish studies found an increased occurrence of larynx cancer in workers exposed to asbestos [7, 8]. The use of asbestos was previously common in the Swedish construction and shipyard industries [31].

### Prevention

The large difference in risk between men and women indicates a greater potential for prevention of larynx cancer in men, such as by avoiding tobacco smoking and possibly decreasing the exposure to occupational air pollutants. However, the decrease in risk by stopping smoking seems to be less than anticipated in Swedish men and later than predicted by IARC [2].

## Conclusion

This ecological study shows the importance of tobacco smoking on the risk of larynx cancer. It indicates that the Swedish pipe smoking may be a significant cause of larynx cancer and an explanation for the difference in rates between men and women. However, pollutants other than tobacco smoking may have an impact on the occurrence of larynx cancer in Sweden. Occupational exposure to air pollutants such as asbestos may also be a cause of the difference in risk between Swedish men and women. The benefits of quitting smoking in relation to the risk of larynx cancer seem to occur later than previously predicted.

## Supplemental Material

sj-docx-1-sjp-10.1177_14034948251327872 – Supplemental material for Smoking and the occurrence of larynx cancer in Sweden – a population analysis Supplemental material, sj-docx-1-sjp-10.1177_14034948251327872 for Smoking and the occurrence of larynx cancer in Sweden – a population analysis by Bengt Järvholm, Per Liv, Linnea Hedman, Maréne Landström, Kjell Torén and Alex Burdorf in Scandinavian Journal of Public Health
