# Supplementary material for: Smoking and the occurrence of larynx cancer in Sweden – a population analysis
Source: Scand J Public Health. 2025 Mar 21;54(2):157–63. doi: 10.1177/14034948251327872 (PMC12936125; doi:10.1177/14034948251327872)
Supplement: sj-docx-1-sjp-10.1177_14034948251327872 – Supplemental material for Smoking and the occurrence of larynx cancer in Sweden – a population analysis [file sj-docx-1-sjp-10.1177_14034948251327872.docx]

Supplement

Table S1. Time trends of incidence rates of larynx cancer between 1970 and 2022, according to sex and age-group.

| Age-group | Men | Women |
| --- | --- | --- |
| 50-54 | -0.023^a^ (-0.029 - -0.018) | -0.015 (-0.028 - -0.003) |
| 55-59 | -0.021 (-0.025 - -0.017) | -0.002 (-0.012 - 0.008) |
| 60-64 | -0.014 (-0.018 - -0.011) | 0.003 (-0.006 - 0.013) |
| 65-69 | -0.014 (-0.017 - -0.011) | 0.024 (0.014 - 0.033) |
| 60-74 | -0.013 (-0.016 - -0.009) | 0.018 (0.009 - 0.028) |
| 75-79 | -0.010 (-0.014 - -0.006) | 0.014 (0.003 - 0.024) |
| 80-84 | -0.004 (-0.009 - 0.001) | 0.003 (-0.012 - 0.018) |

a/ Slope (constant A, see methods) and 95% confidence intervals in parenthesis.

Table S2 Comparison of incidence rates (Relative risk) between men and women.

|  | Year | | |
| --- | --- | --- | --- |
|  | 1972  (1970-1974)^a^ | 1980  (1978-1982) | 1990  (1988-1992) |
| Age-group | RR (95% CI)^b^ | RR (95% CI) | RR (95% CI) |
| 50-54 | 6.4 (3.3–12.5) | 6.3 (3.2-12.3) | 3.6 (2.0-6.4) |
| 55-59 | 10.3 (5.4-19.6) | 7.3 (4.5-12.0) | 6.9 (4.0-11.9) |
| 60-64 | 12.2 (6.9-21.5) | 7.2 (4.6-11.4) | 7.0 (4.4-11.2) |
| 65-69 | 20.4 (10.4-39.8) | 29.5 (13.9-62.8) | 8.6 (5.4-13.5) |
| 70-74 | 18.8 (9.6-36.9) | 18.3 (9.3-35.9) | 11.9 (7.0-20.2) |
| 75-79 | 17.3 (8.0-37.4) | 17.6 (8.9-34.8) | 16.0 (8.6-29.6) |
| 80-84 | 26.1 (6.3-108.7) | 13.9 (6.0-32.3) | 22.0 (9.6-50.5) |

a/ to increase the precision, we studied 5-year intervals.

b/Relative risk; 95% confidence intervals in parenthesis

Table S3. Prevalence of current smokers in older ages according to surveys ( (1-4)).

| **Year** | **Age** | **Men (%)** | **Women (%)** |
| --- | --- | --- | --- |
|  |  |  |  |
| 1980 | 65-74 | 32 | 14 |
| 2000 | 65-74 | 16 | 15 |
| 2004 | 65-84 | 11 | 12 |
| 2021 | 65-84 | 7 | 8 |
|  |  |  |  |
| 1980 | 75-84 | 25 | 4 |
| 2000 | 75-84 | 12 | 9 |

Table S4 Prevalence (%) of current smokers according to age in a national survey in 1963 (N=55,000) (ref (5))

| Age | Men | Women |
| --- | --- | --- |
| 18-24 | 51 | 36 |
| 25-34 | 54 | 32 |
| 35-49 | 53 | 25 |
| 50-69 | 46 | 11 |

Table S5 Time trends of larynx cancer and squamous cell lung cancer.

| Age | Men | |  | Women | |
| --- | --- | --- | --- | --- | --- |
|  | Larynx^a^ | Lung^a^ |  | Larynx | Lung |
| 50-54 | -2.4 (-3.4 - -1.7) | -3.8 (-4.3 - -3.3) |  | -1.5 (-2.8 - -0.3) | -1.0 (-1.7 - -0.4) |
| 55-59 | -2.1 (-2.1 - -1.6) | -3.6 (-4.1 - -3.1) |  | -0.2 (-1.2 – 0.8) | 0.2 (-0.3 – 0.7) |
| 60-64 | -1.5 (-1.9- -1.1) | -2.9 (-3.2 - -2.5) |  | 0.3 (-0.6 – 1.3) | 1.2 (0.8 – 1.5) |
| 65-69 | -1.4 (-1.9 - -1.0) | -2.4 (-2.7 - -2.1) |  | 2.4 (1.4 – 3.3) | 1.8 (1.5 – 2.1) |
| 70-74 | -1.3 (-1.6 - -0.9) | -1.9 (-2.2 - -1.7) |  | 1.8 (0.9 – 2.8) | 2.9 (2.6 - 3.2) |
| 75-79 | -1.0 (-1.4 - -0.6) | -1.5 (-1.8 - -1.3) |  | 1.4 (0.3 – 2.4) | 3.1 (2.8 – 3.4) |
| 80-84 | -0.4 (-0.9 – 0.2) | -1.3 (-1.7 - -0.9) 0.9) |  | 0.3 (-1.2 – 1.8) | 3.2 (2.8 – 3.7) |

.

Change: percent per year; 95% CI in parenthesis. Confidence interval estimated by Wald estimate (Poisson regression for larynx and negative binomial regression for lung cancer due to overdispersion)

Figure SF1. Incidence rates (y-axes, cases per year and 100,000 person-years) for squamous cell lung cancer and squamous cell larynx cancer in men and women according to age and calendar year in 1970-2021. Notice differences in scales on the y-axes.

Figure SF2. Incidence rates (y-axes, cases per year and 100,000 person-years) for adenocarcinoma in lung and squamous cell larynx cancer in men according to age and calendar year between 1970 and 2021. Notice differences in scales on the y-axes.

**References**

1. Ramstedt M. Tobakskonsumtionen i Sverige 2003–2021. [Tobacco consumption in Sweden 2003-2021] Stockhholm: Centralförbundet för alkhol- och narkotikaupplysning, 2022.
2. Tobaksvanor i Sverige 2003-2020. Stockholm: Centralförbundet för alkhol- och narkotikaupplysning, 2021.
3. Tobaksvanor i Sverige. En översikt och analys. (Tobacco habits in Sweden. An overview and analysis.) [Socialstyrelsen redovisar 1986:9]. Stockholm: Socialstyrelsen 1986.
4. Tobaks- och nikotinanvändning – statistik inom området folkhälsa.[Use of tobacco and nicotine – statistics in public health]. <https://www.folkhalsomyndigheten.se/folkhalsorapportering-statistik/statistik-a-o/ovrig-statistik-a-o/tobaksbruk/>. Accessed 2023-01-25.
5. Rökvanor i Sverige; En postenkätndersökning våren 1963 (Smoking habits in Sweden; A mail survey - Sping 1963). Stockholm: Statistiska Centralbyrån, 1965.
